# Do national policies for complaint handling in English hospitals support quality improvement? Lessons from a case study

**DOI:** 10.1177/01410768221098247

**Published:** 2022-05-31

**Authors:** J van Dael, TW Reader, AT Gillespie, L Freise, A Darzi, EK Mayer

**Affiliations:** 1 NIHR Imperial Patient Safety Translational Research Centre, Institute of Global Health Innovation, Imperial College London, London W2 1NY, UK; 2 Department of Psychological and Behavioural Science, London School of Economics, London WC2A 3LJ, UK; 3 Department of Psychology, Bjorknes University, 0456 Oslo, Norway; 4 Department of Surgery & Cancer, Imperial College London, London SW7 2AZ, UK

**Keywords:** patient complaints, National Health Service, England, health policy

## Abstract

**Objectives:**

A range of public inquiries in the English National Health Service have indicated repeating failings in complaint handling, and patients are often left dissatisfied. The complex, bureaucratic nature of complaints systems is often cited as an obstacle to meaningful investigation and learning, but a detailed examination of how such bureaucratic rules, regulations, and infrastructure shape complaint handling, and where change is most needed, remains relatively unexplored. We sought to examine how national policies structure local practices of complaint handling, how they are understood by those responsible for enacting them, and if there are any discrepancies between policies-as-intended and their reality in local practice.

**Design:**

Case study involving staff interviews and documentary analysis.

**Setting:**

A large acute and multi-site NHS Trust in England.

**Participants:**

Clinical, managerial, complaints, and patient advocacy staff involved in complaint handling at the participating NHS Trust (*n*=20).

**Main outcome measures:**

Not applicable.

**Results:**

Findings illustrate four areas of practice where national policies and regulations can have adverse consequences within local practices, and partly function to undermine an improvement-focused approach to complaints. These include muddled routes for raising formal complaints, investigative procedures structured to scrutinize the ‘validity’ of complaints, futile data collection systems, and adverse incentives and workarounds resulting from bureaucratic performance targets.

**Conclusion:**

This study demonstrates how national policies and regulations for complaint handling can impede, rather than promote, quality improvement in local settings. Accordingly, we propose a number of necessary reforms, including patient involvement in complaints investigations, the establishment of independent investigation bodies, and more meaningful data analysis strategies to uncover and address systemic causes behind recurring complaints.

## Introduction

Patient and family complaints (hereinafter: complaints) are increasingly recognised as a critical source of insight for quality improvement. Representing complex narratives of healthcare failures, complaints include social, institutional and clinical problems not always identified by hospital-driven monitoring systems (e.g., incident reporting systems, case reviews),^1, 2^ and have been associated with hospital mortality rates and adverse surgical outcomes.^3, 4^ Critically, most patients and families submit complaints to prevent harm from occurring to others,^5^ but are currently often left dissatisfied.^6, 7^

In the English National Health Service (NHS), which receives over 200,000 formal complaints per year, failures to detect and respond to harm and negligence reported in complaints have been illustrated across a range of public inquiries (e.g., The Mid-Staffordshire Inquiry, The Shipman Inquiry, Morecambe Bay Investigation).^8–10^ In acknowledgement of these failures, several reforms were introduced to improve learning from complaints, such as the regulatory requirements for hospitals to formally investigate and collect data from complaints. Yet, as the most recent Inquiry at The Shrewsbury and Telford Hospital NHS Trust has unfolded, it appears that system-wide progress has been limited (Table 1).

**Table 1.** Key inquiries and policy reviews indicating failings in learning from complaints in English NHS hospitals.

Year	Inquiry or review	Purpose	Key findings relating to failings in the complaints process
2013	A review into the quality of care and treatment provided by 14 hospital trusts in England	Review into the quality of care and treatment provided by 14 English NHS hospital Trusts with persistently high mortality rates.	‘There was a tendency in some of the hospitals to view complaints as something to be managed, focusing on the production of a carefully-worded letter responding to the patient’s concerns as the main output … [over] using that insight to make improvements to services.’ (p.19)^11^
2013	The Mid Staffordshire NHS Foundation Trust Public Inquiry	Investigation into failings and negligence at the Mid-Staffordshire NHS Foundation Trust between 2005 and 2009.	‘Although the complaints of individuals were many in number, and provided graphic proof that something was seriously wrong at the Trust, the complaints were received into a system that failed to draw the necessary alarm signals from them, let alone the relevant lessons.’ (pp. 245–246)^8^
2013	A review of the NHS hospitals complaints system	A review into the handling of complaints in NHS hospital care in England following findings from the Francis Inquiry; mainly through 2500 comments submitted by the public.	‘Many people who complained felt that nothing had been learnt or achieved as a result of their complaint. They were disappointed about this because this had been one of their reasons for complaining in the first place.’ (p. 23)^12^
2015	The Morecambe Bay Investigation	Inquiry into avoidable deaths of at least 11 babies and a mother at Furness general hospital between 2004 and 2013.	‘Reporting to the Board was minimal, focusing on numbers and completion rates within specified days … giving very little indication of what was being complained about, and nothing about actions being taken to rectify issues raised.’ (p. 76)^9^
2017	A review into the quality of NHS complaints investigations	A Parliamentary and Health Service Ombudsman review of 150 NHS investigations in which avoidable harm or death had been alleged in complaints from patients and families.	‘NHS Trusts are not always identifying patient safety incidents and are sometimes failing to recognise serious incidents. When investigations [of complaints] do happen, the quality is inconsistent, often failing to get to the heart of what has gone wrong and to ensure lessons are learnt.’ (p. 2)^13^
2022	Independent review of maternity services at the Shrewsbury and Telford Hospital NHS Trust	A review into maternity failings at The Shrewsbury and Telford Hospital NHS Trust between 2000 and 2019 which initially involved 23 cases of alleged failings, but has since grown to the investigation of 1486 cases.	‘There was a lack of input from senior members of the leadership team in the writing, review, approval, quality control and trend analysis of complaints. … The review team has identified families where care was sub-optimal, where different management would likely have made a difference to the outcome, however the complaint responses justified actions, delays and omissions in care.’ (p. 44)^14^

NHS: National Health Service.

The complex, bureaucratic nature of the NHS complaints system is often cited as an obstacle to effective complaint handling, but a detailed examination of how such bureaucratic rules, regulations and infrastructure shape complaint handling, investigation and monitoring within institutions has yet to be conducted. This study sought to examine how national policies structure local practices of complaint handling, and how are they understood by those responsible for enacting them within local practice.

## Methods

### Study setting

This study was conducted at a multi-site acute NHS Trust in London (England) which consists of five acute sites and a range of community services. The trust was selected based on convenience. The lead researcher was located at the Trust, but had limited pre-existing relationships with the complaints department or frontline. The most recent 2018 Care Quality Commission inspection report at the time of study described the Trust as treating complaints seriously and deriving lessons from investigations. The site was therefore considered an ‘information-rich case’^15^ to explore complaint handling, relative to existing evidence that is mainly generated in poor performing hospitals through public inquiries. A distinctive feature of this Trust is the presence of a centralised complaints department with designated non-clinical ‘investigators’, who occupy a certain degree of distance from frontline practice. The Trust is one of the largest in the country, with an average of over 1,000 complaints per year between 2015 and 2019.

### Participants

Staff were recruited using purposive sampling supported by the complaints manager and frontline contacts. This enabled the identification of relevant staff roles with systematic involvement in complaint handling or with direct experience of receiving a complaint (Table 2). Efforts were made to recruit across different levels of seniority, service types and sites within the Trust. The number of participants per staff group reflects their relative degree of involvement in complaint handling.

**Table 2.** Description of participants by staff group.

Staff group	Description	N
Complaints manager	Oversees complaint handling by screening complaints at initial receipt, reviewing responses and developing quality monitoring reports	1
Complaints administrators	Coordinate complaint handling process by logging details of complaints, supporting investigators and providing point-of-contact to complainants	4
Complaints investigators	Responsible for investigating formal complaints through collaborating with front-line clinical staff to identify what happened, whether the complaint is to be (partly) uphold, and to indicate if there is a need for improvement	3
Clinical managers	Oversee formal complaint investigations on their ward (e.g., provision of staff statements on reported incidents)	5
Patient Advice and Liaison Service	Point-of-contact in the hospital setting to provide advice to patients, resolve informal concerns and receive compliments	3
Local complaints advocacy	Local advocacy service that provides support to complainants who experience difficulty in accessing or going through the complaints process	2
Patient Experience Directorate	Oversee complaints, PALS and other patient feedback activities (e.g., Friends and Family Test, NHS Choices, national surveys)	1
Clinical staff	Front-line staff with experience of having been involved in a complaints case (i.e., no systematic involvement in the complaints process)	1
Total		20

PALS: Patient Advice and Liaison Service.

### Procedure

Semi-structured interviews were held at the organisation’s main hospital between June 2018 and June 2019, lasting an average of 43 minutes (range 10–81 minutes). Interviews were shorter when interviewees had limited regular involvement in the complaints process (e.g., front-line clinical staff). Questions explored staff understandings of how complaints handling routine is enacted. Inconsistencies, workarounds and adverse impacts were explored through follow-up questions, such as through using alternative representations (‘interesting, staff member X said Y’) and problem prompts (‘what happens if [unexpected problem]?’).^16^ The topic guide was developed based on informal observations, document analysis and scoping of existing literature on complaint handling. Informal observations included five hours of shadowing, attending meetings in the complaints department, and informal conversations with the complaints manager and advocacy service. Document analysis included a review of national regulation and policy reports, organisational complaints policy and workflow charts, and hospital records.

### Data analysis

Interviews were audio-recorded and transcribed verbatim. Data were analysed thematically by the lead researcher (JD, social scientist). Open codes were initially developed based on transcripts and documentation, which were then grouped into higher-order organising themes.^17^ A sample of four interviews was also coded by a second researcher (LF, health policy researcher) and discussed to refine codes and interpretations. Interviews were analysed concurrently with the data collection, and alongside documentary analysis, to enable exploration of inconsistencies and to probe emerging themes in subsequent interviews. A process map was developed to describe the routine for handling a complaint as understood by those responsible for enacting it (derived from the interviews with supporting material from national regulatory and local policy documentation to guide interpretation).

## Results

Triangulation of policy documentation and interview transcripts identified four critical areas of practice where the design of national rules and policies functioned to undermine a patient-centric and improvement-focused approach to complaints, relating to access, the conduct of investigations, data collection systems and performance targets. A detailed map of the organisational routine for handling a complaint as described by interviewees can be found in Online supplementary file 1.

### Access: muddled routes for raising concerns

A frequently mentioned issue across staff groups was the confusing landscape of routes for raising concerns. Central to this was the lack of awareness, among both patients and frontline staff, regarding the distinct functions of formal complaints and the Patient Advice and Liaison Service (PALS), a point-of-contact within hospitals created to resolve lower-level concerns and queries directly on the ward. The visibility of PALS (one of its main attributes) positions the services as a catch-all destination for patient concerns and queries, and served to overshadow complaints departments in some cases.

One of the biggest challenges that patients face in contacting us is knowing the difference between informal and formal complaints. They automatically go to PALS because it is there in the hospital, easy to see, and they think that they can help them to make a formal complaint. So, trying to distinguish the difference is something people are really struggling with and they come to us and say ‘I have been to complaints’, but they have not, they have been to PALS. (Patient advocacy worker)

Confusion among clinical staff was evident in the interviews, where some participants repeatedly confused ‘PALS’ with ‘complaints’. Others noted that PALS had become somewhat misused by front-line staff when encountering dissatisfied patients, as reflected in the organisational mantra ‘if unhappy, send to PALS!’ (Clinical manager) referred to by several participants.

Honestly, everyone automatically goes: ‘PALS, if you want to make a complaint, you go to PALS’. I used to do it. I used to work in the booking office. All I knew was, ‘If you want to make a complaint, you go to PALS’. (PALS officer)

The combination of muddled procedures to raise concerns and staff signposting meant that most concerns were handled via PALS, with patients at times unaware that they had not, in fact, complained formally. Although this was positively regarded by hospital staff as providing quick relief to what by some was characterised as a mere ‘failure in interpersonal communication’, it concerned patient advocacy workers who noted that in many cases patients desire the more bureaucratic process because they want their complaint to be formally ‘known and recorded’.

### Investigation: scrutiny, corroboration and defensive tactics

Formal investigative procedures at the Trust were predominantly structured to judge the ‘well-foundedness’ of complaints, as stipulated by national regulations. The legitimacy of complaints was appraised by investigators through cross-validating raised issues with corresponding hospital documentation and staff statements, with internal evidence being regarded as superior.

That is really the key for our investigations, is to make sure there has been some learning. Unless, of course, it is completely unwarranted, the complaint, in which case we will be very direct about that and say, ‘sorry, there is no root to this complaint, and it is well documented that this did not happen.’ (Complaints investigator)

Paradoxically therefore, complaints were only utilised for quality improvement in cases where they described the already known and managed. This reflects a persistent belief that complaints are subjective and subordinate to clinical perspectives and hospital data. It further positions the provider and patient perspectives as antagonistic, with any inconsistency leading to the dismissal of one account, rather than seeking to understand and explore dissonance and realising its potential to reveal institutional blind spots or failures in communication.

If the complainant's recollection is different, mainly different from what you have actually ascertained yourself, then I would say that was not upheld, because our opinion is completely different from theirs. Even though they’re stating that harm was done. (Complaints investigator)

This asymmetric weighting of provider and patient evidence in investigations was further reflected in the comparatively limited opportunities for patients to provide input. Apart from highly sensitive cases, such as those involving death, it was not routine practice to involve complainants in investigations. This stood in stark contrast with opportunities for the involved ward, for whom the investigative process often was described as a highly interactive process between the investigator and the involved ward. One notable exception was a clinical manager of a small ward who had initiated a dialogical practice, where every complaint case was discussed with all actors involved. It was noted that this was made possible by their low case and complaints load, and would be harder to realise in large, busy wards that deal with complaints regularly.

In some cases, the ability for involved staff to shape investigations started long before the investigation. Accounts from investigators described a tendency on the frontline to pre-emptively report detailed accounts of incidents when expecting a complaint.

When the staff realise, I think, on the ward, that a family could possibly put a complaint in, whether warranted or otherwise, they tend then to start to document very detailed summaries of the care. It is very unusual for you to send a complaint through, and the ward not to be expecting it. From that moment on, really, they make sure that everything is documented correctly. (Complaints investigator)

Although most time and resources in the complaints process were spent on investigative activities, only a small proportion of complaints resulted in recommendations for local action, such as a staff re-training, protocol implementation, or policy change (i.e., 4.4% according to hospital records, of which 89.3% were [partly] upheld). Importantly, even in those cases, complaints staff noted it was difficult to close-the-loop and establish whether changes had actually been actioned by staff on the ward.

I am chasing seven actions right now that have not been done, or they might be done in real-life, but they have not been closed on Datix. I have chased most of them three times. (Complaints administrator)

Complaints staff attributed this lack of timely action to an avoidant and defensive attitude towards complaints on the frontline, contributing to their sense of being othered within the institution.

If people did not view complaints as such a negative thing, if there was not a mindset of ‘us’ versus ‘them’ when it comes to people working with us, it would make things a lot easier. Because people just are not overly cooperative at times which can be frustrating because we it is like ‘We work for the same Trust. We are on the same team. Why?’ We are trying to take the negative and make it positive. (Complaints administrator)

### National data collection systems: creating ‘false information’

Although a national data collection system (named ‘KO41a’) was introduced in response to the Mid-Staffordshire Inquiry to ‘improve the patient experience by listening to public voice’,^18^ all four complaints administrators responsible for enacting coding through this scheme considered it inappropriate for use. They consistently referred to the issue that categories did not describe the problems that complaints tend to report and were further insufficiently granular for actionable learning. Two complaints administrators provided the example of a single category to reflect all issues related to clinical care.

You will have a whole load of Clinical Treatment, Clinical Treatment, but you are thinking ‘it is not the Clinical Treatment’. It is not broken down correctly at all. For me, I see it as false information. It is not accurate so, therefore, how can you know how to improve? (Complaints administrator)

As this taxonomy represented the main means for reporting on trends across complaints at national and organisational levels, this resulted in scepticism regarding the usefulness of these reports for quality monitoring and improvement.

I know that [the complaints manager] will run reports from the hospital’s informatics system and pull out the trends, so he will see how many complaints were logged, for example, under Clinical Treatment. So, yes, he will say, ‘Okay, 80 per cent of my complaints'. I do not know what he does with that information because that cannot be useful. (Complaints administrator)

These limitations resulted in data entry merely being perceived as a ‘tick box exercise’, despite representing a large portion of time and work involved in complaint handling. Within a system already short in time and resources, there was a sense that time spent coding could better be used for interacting with patients and providing social support.

Unsurprisingly, the data collection system did not adequately support the complaints manager in identifying recurring themes across complaints, who was necessitated to rely on memory rather than recorded data. Accordingly, the complaints manager noted the need for a ‘smarter’ system to record and monitor incoming complaints.

To see trends, see emerging themes, perhaps things that I might not have been able to spot. I think that would be really good, because often we are relying on our feel for it, but if there was a way to flag up – ‘you’ve had five about this in the last week’ – it would be really good. (Complaints manager)

The importance of logging and identifying recurring problems was echoed by clinical managers and a complaints investigator, who noted that sole reliance on case-by-case investigations provides limited means to understand whether there are systemic factors behind local issues.

I think we probably should do more following up and trying to gauge whether there are similarities across areas and whether there is deeper learning that we can take from the complainants. Because I think we probably do the learning from an individual complaint in an individual department reasonably well, but does that ripple out further? I am not sure we follow up a lot with: ‘are there similarities between these and does that reveal a bigger need?'. (Complaints investigator)

### Performance targets, adverse incentives and workarounds

At managerial levels, monitoring relating to complaints was primarily focused on national performance targets for complaints handling, which in turn are mainly related to timescales for investigating and responding to complainants, and volumes of complaints received, leaving their relative severity unexplored.

The Trust like numbers because it is easier to get your head around than outcome targets. This year we have had something like 50 fewer complaints than last year, so that is a good thing because it shows we are getting better. But it does not tell you that actually the complexity and severity of some of the complaints this year were beyond anything we have ever seen before. (Patient Experience Directorate)

One interviewee expressed concern about the focus on reducing complaints volumes as creating adverse incentives, such as impeding accessibility of the complaints process, as reflected in a statement provided by one of the interviewees ‘we want PALS to go up and complaints to go down’ (PALS officer), which may partly explain frequent signposting to PALS as discussed in theme 1.

This year we have got number targets which I am in two minds about … if you've got a reduction in formal complaints, it could suggest that actually our care is getting better and people have less reason to complain. It could, however, indicate that we don't have a very open culture and we're suppressing complaints, so we could be saying we'll just pass this one on to someone else or we'll have people in the divisions discouraging people from raising concerns. (Patient Experience Directorate)

Performance targets for complaint handling predominantly focused on administrative aspects, with pressure not to exceed response timelines set out by national policy. The influence of these targets on staff sensemaking of their role and goals was evident in the interviews. For example, following current policy, the number of days that hospitals have to complete an investigation is dependent on the complaint’s relative level of risk. This contingency between time and risk meant that risk ratings had become operationalised as a mechanism to manage the often pressured timelines of investigations (rather than purely an indicator for level of risk).

So let’s say, it’s a joint complaint with different trusts, that automatically goes as medium risk because they need their time and we need our time to get our details straight. (Complaints coordinator)

The normalisation of this workaround was reflected in staff accounts when asked how they understood ‘risk’.

Medium is 45 [days to investigate], and high is 65. … It’s more about time. That’s how I’d see it, now. Obviously, if there is a very serious complaint, of course it’s going to be medium, but it’s just more about time. (Complaints administrator)

## Discussion

Our study contributes to existing complaint handling research by illuminating how national policy can shape local practices and can impede an improvement-focused approach to complaints.^19–22^ The procedural problems identified in our findings speak to a recent complaints study in the English NHS which concluded that failures in learning are not necessarily ‘a consequence of sinister or malign organisational actors seeking to impose silence’ (p. 7)^23^, and, instead, can be a case of (often) well-intentioned staff confined by an overly formalised and bureaucratic system. Through a detailed examination of the enactment of this system within local practice, we have generated a number of recommendations for reform (Table 3).

**Table 3.** Lessons and recommendations for the NHS complaints process based on this study’s findings.

1. Clarify the distinct roles of PALS and formal complaints processes to staff and patients, such as through leaflets and signposting within hospitals, to avoid PALS from being a barrier to the formal process. (theme 1)
2. Remove the regulatory requirement for hospitals to judge whether complaints are ‘well-founded’. All complaints are opportunities towards better understanding patients’ needs and their unique perspective on organisational safety. Involve patients and families in complaints investigations as standard practice and create opportunity for dialogue between involved staff and harmed patients. (theme 2)
3. Establish independent complaints bodies for investigating and analysing complaints in order to fully leverage the potential of complaints to flag problems that risk being ignored, contested, or underappreciated through institutional sensemaking frames (in particular in settings with poor safety culture or stigma around complaints). (theme 2)
4. Improve or replace national data collection systems (i.e., ‘KO41a’) which currently represent a bulk of time and effort involved in complaint handling, but produce meaningless results. A reporting taxonomy needs to sufficiently discriminative to distinguish patterns of poor care and support the triaging of deeper investigation. A taxonomy should also have construct validity: i.e., reflect the themes patients describe in complaints (rather than the categories that policy makers and providers wish to count and manage). (theme 3)
5. Ensure that administrative and quantitative Key Performance Indicators for complaint handling (e.g., time to respond, numbers received) are not prioritised over harder-to-measure outcomes, such as those regarding learning and improvement. Timely responses are important for complainants, but should not be at cost of efforts to improve. Similarly, the monitoring of simple numbers of complaints as a quality indicator is inappropriate, as it does not provide information about the severity or complexity of complaints – e.g., a small number of complaints can indicate an inaccessible process and the tip of an iceberg, rather than high-quality care. (theme 4)

NHS: National Health Service; PALS: Patient Advice and Liaison Service.

Unlike countries with (semi-)independent complaints bodies (e.g., Finland, Sweden), English settings are required to investigate their received complaints, and report whether they are ‘well-founded’^24^. Although, in theory, local investigations enable hospitals to action immediate improvements, our study suggests this may only occur for the small proportion of complaints that are corroborated by internal points of view, or already part of existing quality improvement workstreams, and thus reflect the already known and managed. This serves not only to uphold unequal power dynamics through assuming the superiority of clinical perspectives, but also negates the precise value of complaints as a means to uncover problems that tend to be missed, discounted or underappreciated by those within institutions. Unsafe or poor practices in healthcare often reflect issues that are normalised and thus, to some extent, blind to those enacting them.^25^ Dissonant, outsider perspectives, such as those captured in complaints, are needed to highlight and challenge these practices.^2,26^

Further, asking hospitals to grade their own homework carries particular risks in the context of organisations with poor safety culture. The impact of a hospital’s shared norms, values and beliefs on the effectiveness of safety practices is well known in the case of incident reporting systems and safety investigations,^27, 28^ and may have similar effects on a hospital’s conduct of complaints investigations – meaning complaints mechanisms may be least effective in settings where they are most needed.

Although national efforts have been made to improve learning through national data collection systems (e.g. ‘KO41a’^18^), this did not generate meaningful quality monitoring outputs at the investigated setting. This is in sharp contrast to the growing body of research that has developed and validated methods to reliably analyse complaints.^29^ Regardless, it can be argued that narrative and dialogical approaches that enable the juxtaposition of sensemaking between patients and providers, such as patient involvement in investigations, listening clinics or public committees, may offer greater potential in understanding the needs and experiences of patients, and uncovering the implicit assumptions, beliefs and practices that make organisations unsafe.

### Study strengths and limitations

Although findings resonate with earlier reviews at other English NHS settings,^8, 12^ it must be noted that this study was conducted at a single multi-site NHS organisation, meaning that the findings cannot be assumed to be generalisable across settings or countries. To aid interpretation of findings relative to other settings, a detailed description of the study setting was included. A strength of the case study design was that it allowed for an in-depth exploration of enactments and adverse impacts of national policies in local practice.^30^

Critically, ‘work-as-imagined’ often varies from ‘work-as-done’.^31^ We aimed to gain insight on the latter by querying the activities of staff (‘what do you do?’, ‘what do you do next?’, ‘and then?’), triangulation with policy documentation, problem prompts and alternative representations. However, given that the study predominantly relied on interviews, the data represent a mix of how staff envision they are required to conduct the work and how this can play out in different ways, and we acknowledge that the study would have benefited from direct observations.

## Conclusion

This study has contributed to existing evidence by demonstrating how challenges to translating complaints into quality improvement can originate from nationally defined policies and regulations for complaint handling. Recommendations for change include patient involvement in complaints investigations, the establishment of independent investigation bodies, and more meaningful data analysis strategies to uncover and address systemic causes behind recurring complaints at national and organisational levels.

## Supplemental Material

sj-pdf-1-jrs-10.1177_01410768221098247 - Supplemental material for Do national policies for complaint handling in English hospitals support quality improvement? Lessons from a case study Click here for additional data file. Supplemental material, sj-pdf-1-jrs-10.1177_01410768221098247 for Do national policies for complaint handling in English hospitals support quality improvement? Lessons from a case study by J van Dael, TW Reader, AT Gillespie, L Freise, A Darzi and EK Mayer in Journal of the Royal Society of Medicine
