# Supplementary material for: Do national policies for complaint handling in English hospitals support quality improvement? Lessons from a case study
Source: J R Soc Med. 2022 May 31;115(10):390–8. doi: 10.1177/01410768221098247 (PMC9720291; doi:10.1177/01410768221098247)
Supplement: sj-pdf-1-jrs-10.1177_01410768221098247 - Supplemental material for Do national policies for complaint handling in English hospitals support quality improvement? Lessons from a case study [file sj-pdf-1-jrs-10.1177_01410768221098247.pdf]

Online supplemental file 1: Organisational routine for complaint handling

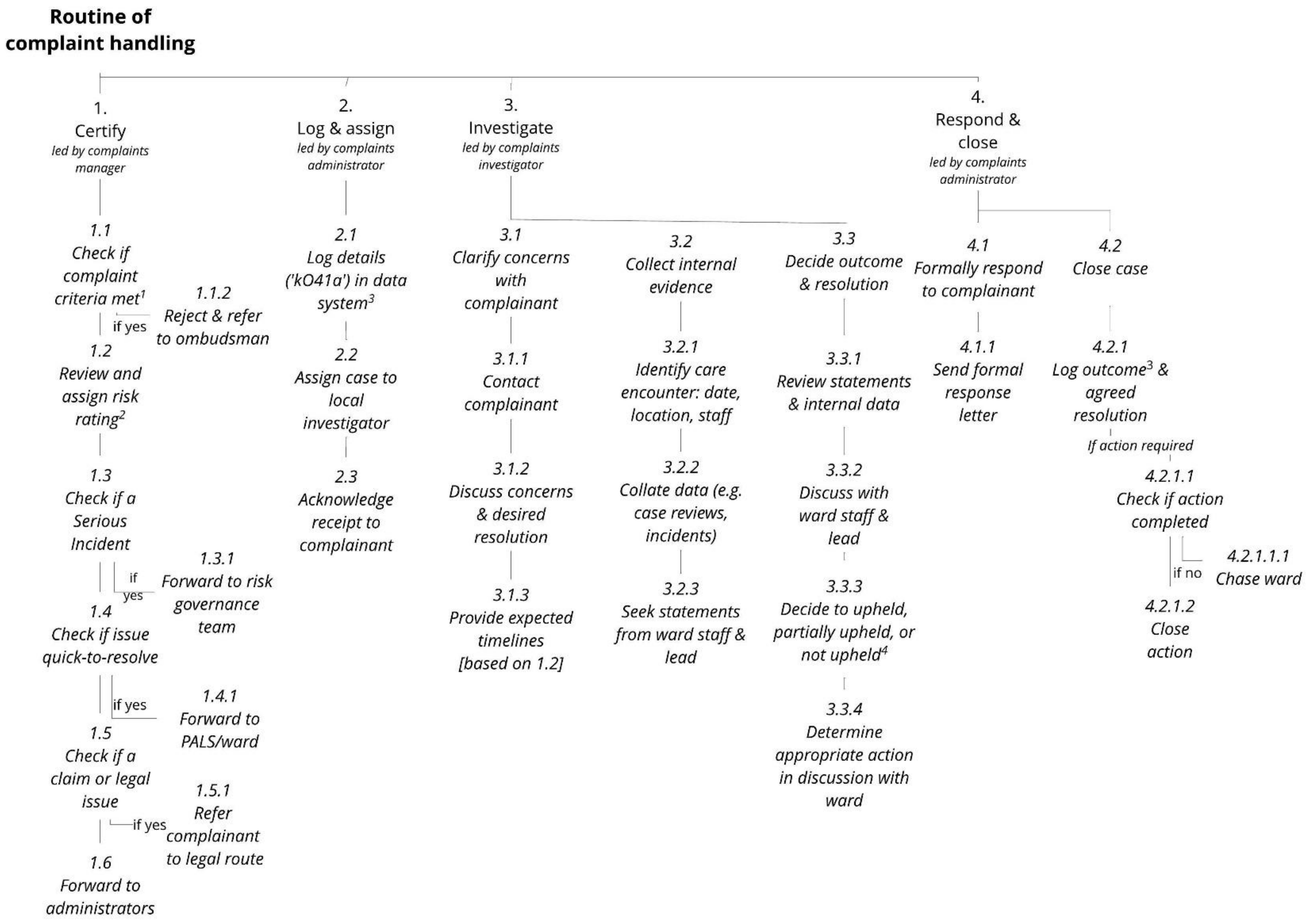

[1]The complaint should be submitted within 12 months of incident giving rise to complaint.
[2]The assigned "risk" rating determines the number of days the hospital has to respond: 25 working days for *low*, 45 working days for *medium*, 65 working days days for *high*. This meant that , in practice, risk was operationalised as an indicator for case complexity (e.g., a multi-site complaint would be assigned *medium* or *high* as it requires more time to investigate), rather than safety risk or severity.
[3]Collected details are guided by the national "KO41a" collection, which include: subject area of complaint; patient age, status of complainant (e.g., relative, patient, or solicitor), service area, profession complained about, and investigation outcome.
